# Molecular Role of Ca^2+^ and Hard Divalent Metal Cations on Accelerated Fibrillation and Interfibrillar Aggregation of α-Synuclein

**DOI:** 10.1038/s41598-018-20320-5

**Published:** 2018-01-30

**Authors:** Jong Yoon Han, Tae Su Choi, Hugh I. Kim

**Affiliations:** Department of Chemistry, Korea University, Seoul, 02841 Republic of Korea

## Abstract

α-Synuclein (αSyn) is an intrinsically disordered protein, the aggregation of which is highly related to the pathology of diverse α-synucleinopathies. Various hard divalent metal cations have been shown to affect αSyn aggregation. Especially, Ca^2+^ is suggested to be a crucial ion due to its physiological relevance to α-synucleinopathies. However, the molecular origin of αSyn aggregation mediated by the metal ions is not fully elucidated. In this study, we revealed that hard divalent metal ions had almost identical influences on αSyn aggregation. Based on these similarities, the molecular role of Ca^2+^ was investigated as a representative metal ion. Herein, we demonstrated that binding of multiple Ca^2+^ ions induces structural transition of αSyn monomers to extended conformations, which promotes rapid αSyn fibrillation. Additionally, we observed that Ca^2+^ induced further interfibrillar aggregation *via* electrostatic and hydrophobic interactions. Our results from multiple biophysical methods, including ion mobility-mass spectrometry (IM-MS), synchrotron small-angle X-ray scattering (SAXS), transmission electron microscopy (TEM), provide detailed information on the structural change of αSyn and the aggregation process mediated by Ca^2+^. Overall, our study would be valuable for understanding the influence of Ca^2+^ on the aggregation of αSyn during the pathogenesis of α-synucleinopathies.

## Introduction

A number of neurodegenerative diseases, including Alzheimer’s disease, Parkinson’s disease, and Huntington’s disease, are associated with the formation of amyloid fibrils^1^. During the fibrillation of amyloidogenic proteins, monomeric proteins are converted into oligomeric intermediates, and finally, to highly ordered, unbranched β-sheet structures^2,3^. Amyloid fibrillation is not thoroughly understood yet; however, it is considered to have a correlation with protein misfolding^2,4^. Therefore, understanding the mechanisms of amyloid fibrillation, which is associated with protein misfolding, is necessary for developing therapeutic strategies for amyloidosis.

**Figure 1:**
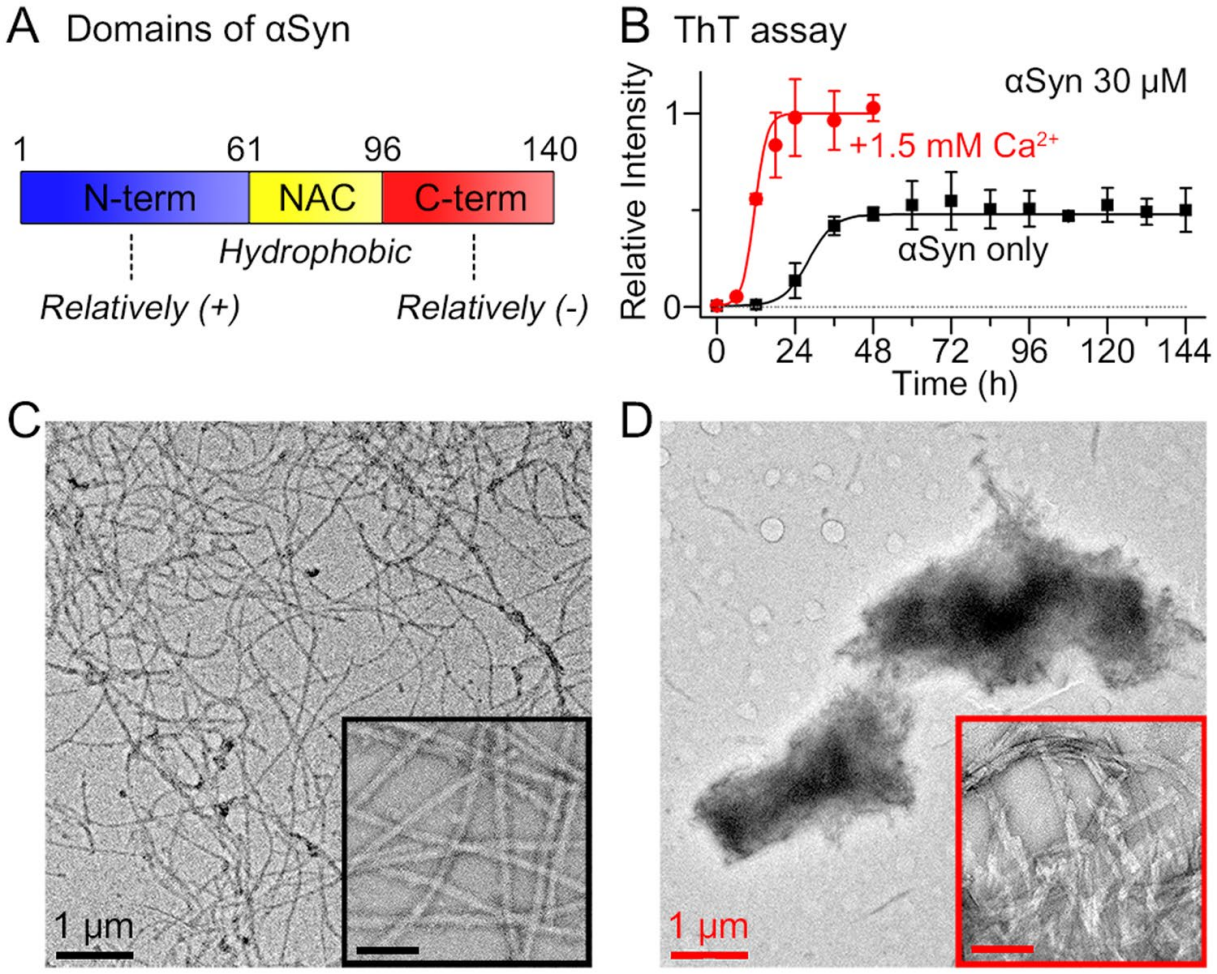
Ca^2+^-mediated αSyn aggregation. (**A**) Three domains of αSyn. (**B**) ThT assay for the fibrillation kinetics of αSyn (30 μM) in the presence and absence of 1.5 mM CaCl_2_. All αSyn samples included a low level of residual Ca^2+^ (~10 μM). (**C**) TEM images of αSyn fibrils formed in the absence of Ca^2+^ and (**D**) aggregates formed in the presence of 1.5 mM Ca^2+^. The scale bars in the insets of TEM images are 100 nm.

α-Synuclein (αSyn) is a small amyloidogenic protein, which is abundant, particularly, at the presynaptic nerve terminals^5–7^. αSyn is considered to modulate the synaptic vesicle cycle, which is involved in neurotransmission^7,8^; however, it has received attention because of its pathological significance as the main component of Lewy bodies and Lewy neurites, observed in patients with α-synucleinopathies such as Parkinson’s disease (PD), multiple system atrophy (MSA), and Lewy body dementia (LBD)^9^. αSyn is an intrinsically disordered protein (IDP), and comprises 140 amino acid residues, which constitute the amphipathic domain at the N-terminal region (residues 1–60), the hydrophobic non-amyloid-β component (NAC) region (residues 61–95), and the acidic domain at the C-terminal region (residues 96–140) (Fig. 1A). The structure of the NAC region in particular, is considered important for fibrillation kinetics^10,11^. In water, the NAC region tends to be located towards the interior of the protein, and is shielded from contact with water^12,13^. This innate structure of αSyn induced by intramolecular interaction hinders intermolecular aggregation. However, once the NAC region is exposed to the outside because of the structural transitions of αSyn, the interface between water and the exposed NAC region induces intermolecular hydrophobic interactions between the NAC regions.

The aggregation of αSyn is believed to be associated with environmental factors, such as an imbalance in metal homeostasis^14,15^. Cu^2+^, a divalent transition metal ion, has been proposed as a potential cause of αSyn fibrillation with its high binding affinity (*K*_d_ ~10^−6^–10^−10^ M) and unique binding site (The N-terminus and His-50 are involved)^16–18^. Unlike Cu^2+^, hard divalent metal cations have been shown to promote the aggregation of αSyn^15,19,20^ with binding to the acidic C-terminal region (*K*_d_ ~ 10^−3^ M)^19,21^. The metal cations also induce the formation of Lewy body-like large assemblies comprising αSyn fibrils^22^. However, the mechanistic details on the metal-associated fast aggregation and Lewy body-like interfibrillar aggregation of αSyn have not been fully understood. Among the hard divalent metal ions, converging evidence suggests that Ca^2+^ is a crucial physiological factor related to αSyn aggregation. Firstly, the dysregulation of Ca^2+^ has been observed in aged animals^23^ and mice model of α-synucleinopathies^24^. Abnormally increased intracellular Ca^2+^ concentration, which is normally regulated to be ~100 nM (while its extracellular concentration is ~1.4 mM)^25^, can cause aggregation of αSyn^26–28^, and finally induce neurodegeneration^26,29^. Secondly, significant amount of Ca^2+^ has been detected in Lewy bodies of patients with PD^30^. This suggests the possibility of the involvement of Ca^2+^ in the formation of Lewy bodies. Furthermore, when αSyn is secreted to the extracellular space (secretion of αSyn is commonly observed in PD model systems^31–33^), αSyn can be exposed to and influenced by high level of Ca^2+^ (~1.4 mM). Taken together, the interaction between αSyn and Ca^2+^ appears to be closely related to αSyn aggregation in α-synucleinopathies.

Herein, we have reported the unique αSyn aggregations mediated by hard divalent cations to form large interfibrillar aggregates. Then, the mechanism of αSyn aggregation mediated by Ca^2+^, a representative hard divalent cation, was proposed by monitoring the structural transition of αSyn from the monomeric state to the large interfibrillar aggregate state. Our structural and kinetic results, which were obtained using multiple biophysical methods, including ion mobility-mass spectrometry (IM-MS), transmission electron microscopy (TEM), synchrotron small-angle X-ray scattering (SAXS), and inductively coupled plasma optical emission spectroscopy (ICP-OES) demonstrated that Ca^2+^ mediates the rapid formation of αSyn fibrils *via* the structural transition of monomeric αSyn into an extended conformation, which is prone to aggregation. We probed that direct interaction between Ca^2+^ and αSyn fibril induces the subsequent association of the fibrils with secondary structure changes to form large interfibrillar αSyn aggregates through electrostatic and hydrophobic interactions. Moreover, we observed that αSyn aggregates formed through Ca^2+^ mediation are toxic to SH-SY5Y neuroblastoma cells. We believe that the aggregation mechanism of αSyn mediated by Ca^2+^ provides an insight into the formation mechanism of the inclusion bodies that is commonly observed in α-synucleinopathies.

## Results and Discussion

### αSyn aggregation mediated by Ca^2+^ and other hard divalent metal cations and morphological properties

First, we investigated the fibrillation kinetics of αSyn in the presence of Ca^2+^ using the thioflavin T (ThT) assay (Fig. 1B). Fibrillation of αSyn was accelerated by the addition of Ca^2+^ (*t*_1/2_ = 11.6 h)^19,27,34^ compared with the control group, αSyn incubated without Ca^2+^ (*t*_1/2_ = 28.1 h). Furthermore, the ThT fluorescence intensity of Ca^2+^-mediated αSyn aggregates was almost twice as high as the control group, which implied that Ca^2+^ promoted the conversion of more monomers to fibrils. Then, the morphology of aggregates was observed using transmission electron microscopy (TEM). In the absence of Ca^2+^, normal amyloid fibrils were formed (Fig. 1C). In contrast, from αSyn incubated with Ca^2+^, micrometer-scale globular αSyn aggregates were formed (Fig. 1D). The inset of Fig. 1D showed that the αSyn aggregates formed through Ca^2+^ mediation are clusters of fibrils, as previously reported by Semerdzhiev *et al.*^22^.

**Figure 2:**
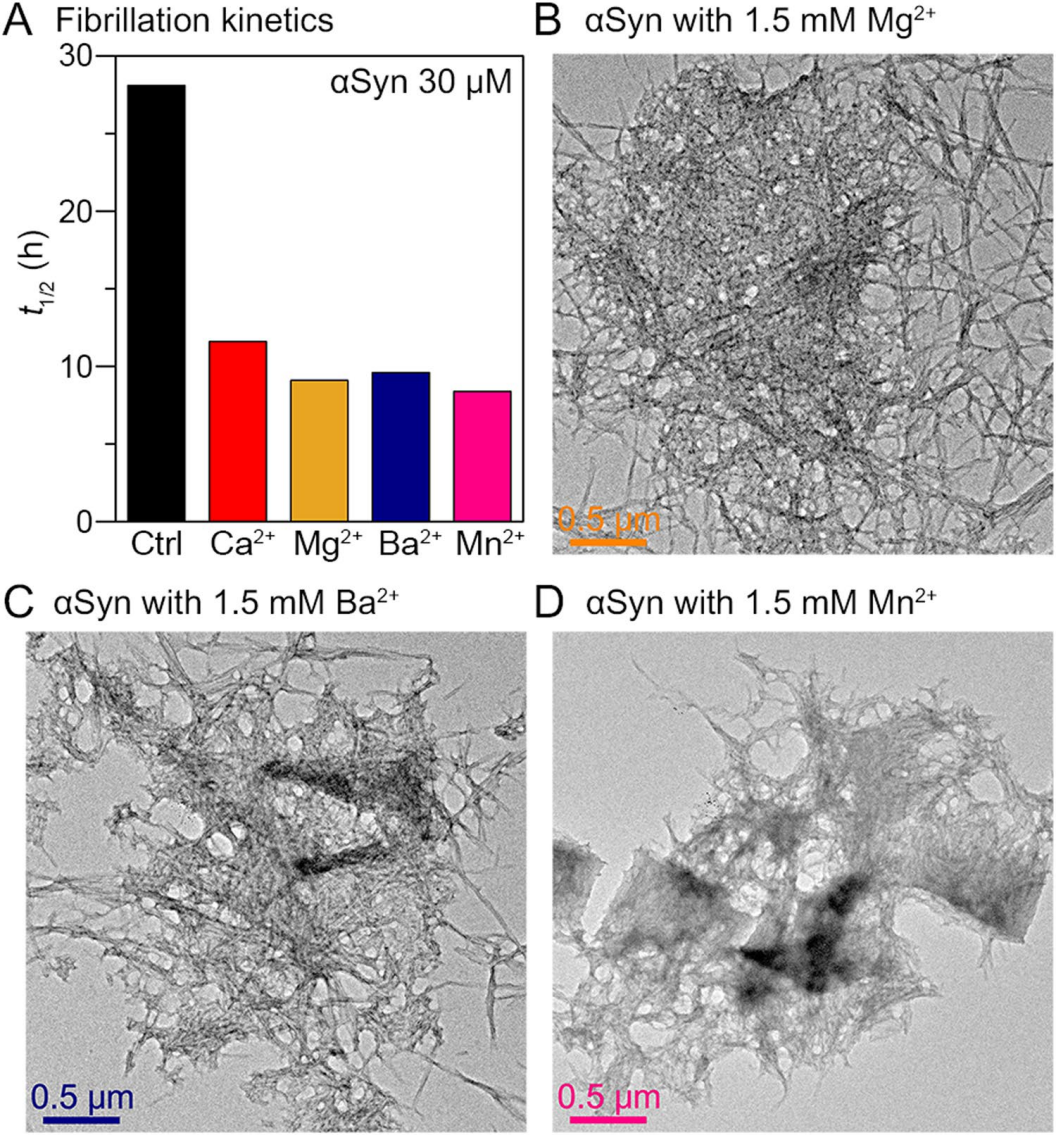
αSyn aggregation mediated by hard divalent metal ions. (**A**) The half-time of fibrillation (*t*_1/2_) of αSyn (30 μM) in the presence of 1.5 mM hard divalent metal ions. The *t*_1/2_ values were obtained by performing ThT assay. TEM images of αSyn aggregates formed in the presence of (**B**) 1.5 mM Mg^2+^, (**C**) 1.5 mM Ba^2+^, and (**D**) 1.5 mM Mn^2+^.

Then, we examined other hard divalent metal ions which bind to the C-terminal region of αSyn, to understand the generality of the metal charge state and the binding site on αSyn fibrillation. For the experiment, Mg^2+^ and Ba^2+^, smaller and larger alkaline-earth metals than Ca^2+^, respectively, and Mn^2+^, as an example of a transition metal, were chosen. Using ThT assay, we observed that these hard divalent metal ions also accelerated the fibrillation of αSyn (Fig. 2A and Supplementary Fig. 1). We found that the metal ions induced the formation of large aggregates comprising fibrils (Fig. 2B–D). These results indicated that hard divalent ions have similar effects to the aggregation of αSyn by their unique complexation with αSyn.

**Figure 3:**
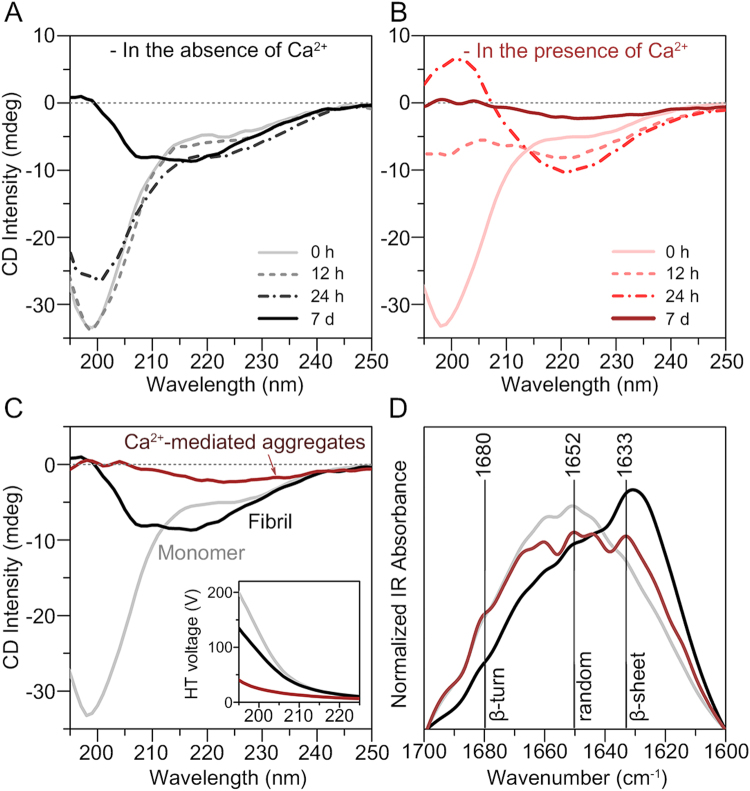
Secondary structural analysis of the αSyn aggregates formed through Ca^2+^ mediation. Time-resolved CD spectra of two-fold diluted αSyn samples (30 μM) incubated (**A**) in the absence of Ca^2+^ and (**B**) in the presence of 1.5 mM Ca^2+^. All incubated αSyn samples included a residual Ca^2+^ concentration of ~10 μM. (**C**) CD spectra and (**D**) IR spectra of the αSyn monomers (gray), fibrils (black), and aggregates formed through Ca^2+^ mediation (brown). The inset in the image of CD spectra shows HT voltage of the samples, which was simultaneously measured during CD measurements.

To understand the formation mechanism of αSyn aggregates by divalent metal cations, secondary structural analysis was performed on Ca^2+^-mediated αSyn aggregates using time-resolved circular dichroism (CD) spectroscopy. The control group without Ca^2+^ exhibited random coil conformation at 0 h; then, the initial conformation was gradually converted to a β-sheet conformation (negative band at 218 nm) after incubation for 7 d (Fig. 3A). In the presence of Ca^2+^, αSyn also exhibited random coil conformation before incubation (Fig. 3B). The conformation of αSyn, which was incubated with Ca^2+^, was rapidly converted to β-sheet conformation at 24 h. However, after incubation for 7 d, the spectrum of the αSyn aggregates formed through Ca^2+^ mediation exhibited distinctive characteristics. This pattern of events implied that Ca^2+^ promotes the formation of β-sheet-rich fibrillar aggregates in the early stage; however, Ca^2+^ mediates a different type of αSyn aggregate in the later stage.

The CD spectrum of the Ca^2+^-mediated αSyn aggregates formed by incubation for 7 d was completely different from that of the conventional αSyn fibrils. αSyn fibrils exhibited a β-sheet-rich structure; however, the CD intensity of the Ca^2+^-mediated αSyn aggregates was low (Fig. 3C). In addition, the intensity of high tension (HT) voltage (Fig. 3C, inset), which is proportional to the absorbance of the samples^35^, was lower in the Ca^2+^-mediated aggregates. The absorbance of large protein aggregates is usually reduced compared with the expected absorbance as calculated using the Beer-Lambert equation, because large aggregates have lower effective cross-sectional area of chromophores than uniformly dissolved samples^36^. To confirm that the observed low CD intensity and HT voltage did not originate from the rapid sedimentation of αSyn aggregates, we measured the sedimentation degree of well-dispersed αSyn fibrils and Ca^2+^-mediated aggregates. The suspensions were left to stand for 0–30 min and the supernatants were analyzed by using ThT assay and optical density (OD) measurements (Supplementary Fig. 2). Our results showed that both rarely sedimented for 5 min, which indicated that the reason for the low CD intensity of Ca^2+^-mediated aggregates was their large size, as shown in Fig. 1D, which did not result from the sedimentation of aggregates during the CD measurement (~5 min).

To further reveal the secondary structure of αSyn aggregates formed through Ca^2+^-mediation, we performed infrared (IR) spectroscopy. The IR spectra indicated that the αSyn aggregates formed through Ca^2+^ mediation consisted of a low structural portion of β-sheets, compared with the conventional fibrils (Fig. 3D). Based on the different morphologies as observed by TEM and secondary structures as observed by IR spectroscopy, it is suggested that the interaction with Ca^2+^ stimulates the formation of distinct αSyn aggregates. Overall, our results, as shown in Figs 1 and 3, suggested that Ca^2+^ promotes the fibrillation of αSyn, and mediates the formation of β-sheet-rich structures in the early stage. Then, Ca^2+^ further induces the formation of large αSyn aggregates through interfibrillar aggregation and secondary structural change.

### Rapid aggregation of αSyn with Ca^2+^-induced structural transition of monomers

**Figure 4:**
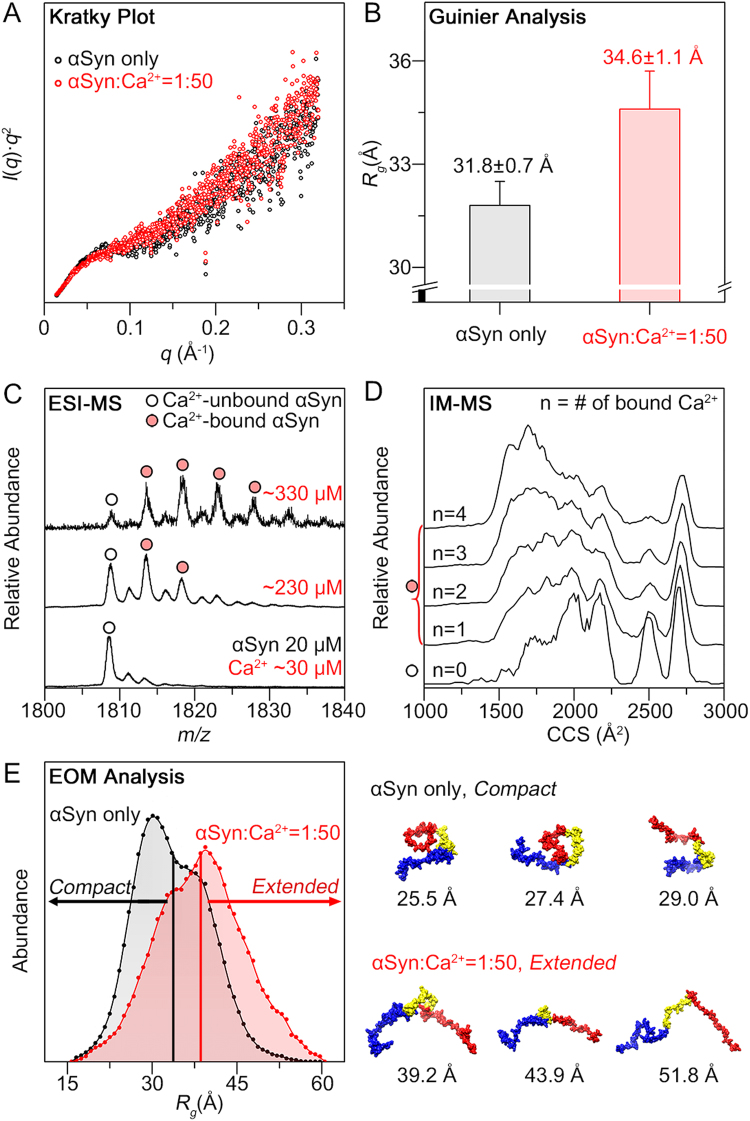
Conformational transition of αSyn monomers induced by Ca^2+^. (**A**) Kratky plots of αSyn (200 μM) in the absence and presence of 50-equivalent Ca^2+^ (10 mM). (**B**) *R*_*g*_ values obtained by the Guinier analysis of SAXS scattering curves. (**C**) ESI-mass spectra of αSyn (20 μM) in the + 8 charge state with increasing Ca^2+^ concentration. The residual Ca^2+^ concentration of the αSyn sample without CaCl_2_ (bottom) was ~30 μM and all Ca^2+^ concentrations were the sum of the residual Ca^2+^ and added CaCl_2_. In the mass spectra, Ca^2+^-unbound and bound αSyn were marked with empty circles and filled circles, respectively. (**D**) IM-MS spectra of αSyn in the + 8 charge state with increasing number (n) of bound Ca^2+^ ions ([αSyn + nCa + (8–2n)H]^8+^). (**E**) *R*_*g*_ distribution of the structural ensemble (50 structures) obtained using EOM and some representative structures of the ensemble with the *R*_*g*_ values. Residues that are shown in blue, yellow, and red indicate the N-terminal, NAC, and C-terminal regions, respectively.

Because αSyn aggregation commonly involves structural transition of monomeric protein^9,37^, we utilized SAXS and IM-MS to characterize the structures of Ca^2+^-bound αSyn monomer. The Kratky analysis (*I*(*q*)·*q*^2^ vs *q*) from the SAXS profiles^38^ showed the typical curves of unfolded proteins (*i.e*. the lack of bell-shaped curves and increase in the *q* range)^38,39^ for both αSyn in the presence and absence of Ca^2+^ (Fig. 4A). However, the values of radius of gyration (*R*_*g*_) of αSyn, which were obtained from the Guinier analysis, showed that *R*_*g*_ increased when Ca^2+^ was added. The *R*_*g*_ value of αSyn was measured to be 31.8 ± 0.7 Å, which was similar to the value obtained in the previous study (Fig. 4B)^40^. As Ca^2+^ concentration in the solution increased, the *R*_*g*_ value of αSyn tended to increase (Supplementary Fig. 3). The *R*_*g*_ values reached 34.6 ± 1.2 Å at 10-fold molar ratio of Ca^2+^ to αSyn, and a similar value was maintained at 50-fold ratio (Fig. 4B and Supplementary Fig. 3). These results indicated that Ca^2+^ induces αSyn monomer to form extended conformations in solution.

IM-MS coupled with electrospray ionization (ESI) detected that multiple Ca^2+^ ions were bound to αSyn monomer (20 μM). As Ca^2+^ concentration increased, the maximum number of Ca^2+^ bound to αSyn and the relative abundance of Ca^2+^-bound αSyn peaks in the mass spectrum increased (Fig. 4C and Supplementary Fig. 4). In particular, a maximum of four Ca^2+^ ions were bound to one αSyn molecule in the presence of 330 μM CaCl_2_. In solution, Ca^2+^ ions bind to multiple binding sites of the C-terminal region with the binding enthalpy (*Δ*H°) ~2 kcal/mol^41^ and weak affinity (*K*_d_ ~ 1 mM)^19,41^. Because four binding sites were previously reported^41^, based on *K*_d_ ~ 1 mM and the exact Ca^2+^ concentration, 0.71 and 0.95 Ca^2+^ per αSyn molecule (230 μM and 330 μM Ca^2+^, respectively) could bind on average. However, the electrostatic interactions between a protein and metal cations can be further enhanced in the gas phase, because solvent molecules evaporate during the ESI process^42^. Thus, despite the low binding affinity, a large number of bound Ca^2+^ ions, which were weakly associated with the C-terminal region *via* long range attractive interaction, were ultimately observed in the gas phase. In addition, the increased number of Ca^2+^ ions bound to αSyn ions in the sample with 330 μM Ca^2+^ than in the sample with 230 μM Ca^2+^ (Fig. 4C) was considered to be related to the charge saturation and location of metal ions in electrosprayed droplets. In the droplet, metal ions are likely located near the surface with higher number density as the metal concentration increases^43^, which can influence the complexation with IDPs, favoring it to be near the surface of the droplet^44^. Furthermore, we found that the total charge of the ESI was saturated at just below 330 μM (Supplementary Fig. 4) in our experimental conditions^45^. As a result, enhanced numbers of Ca^2+^ ions were most likely located at the surface of the droplet to generate αSyn ions, with a maximum of four Ca^2+^ from the sample of 330 μM CaCl_2_.

The IM-MS spectra of Ca^2+^-bound αSyn showed multiple ion mobility peaks with collision cross-section (CCS) values ranging between 1400~2700 Å^2^ for +8 charged αSyn (Fig. 4D). As the number of Ca^2+^ that were bound to αSyn increased, αSyn molecules tended to adopt more compact conformations in the gas phase. This pattern was opposite to the results of the SAXS measurement in solution.

To understand the relation between the structural transitions of αSyn stimulated by Ca^2+^ and its accelerated aggregation, molecular dynamics (MD) simulation of Ca^2+^-bound αSyn was performed to match our SAXS and IM-MS data. Representative structures of αSyn in solution were obtained from the structure pool of αSyn generated using replica exchange MD simulations^46^, based on the ensemble optimization method (EOM), which identifies the best ensemble by fitting sum of multiple theoretical SAXS profiles to the experimental SAXS profile^47,48^. Using the obtained representative structures as initial structures, gas-phase MD simulations were also performed to obtain gas-phase structures having theoretical CCS values (CCS_theo_) corresponding to the experimental values. The CCS_theo_ value of each structure was estimated using the exact hard sphere scattering (EHSS) method^49^, and compared with the experimental CCS values (Supplementary Fig. 5).

The finally obtained αSyn ensembles in the absence and presence of Ca^2+^ in solution (50 structures for each ensemble) showed differences in *R*_*g*_ distributions. The αSyn ensembles that were obtained in the absence of Ca^2+^ and in the presence of Ca^2+^ had distributions ranging from 15 to 60 Å; however, the former had a distribution with an average of 33.6 ± 6.4 Å, while the latter had a distribution with an average of 38.5 ± 7.7 Å (Fig. 4E). Although the difference between averaged *R*_*g*_ values of αSyn is not significant, the *R*_*g*_ distribution of 50 αSyn structures showed the clear trend that the abundance of extended conformations was increased in the presence of Ca^2+^. The representative structures of the extended conformations of αSyn with *R*_*g*_ > 38.5 Å showed that the hydrophobic NAC region was generally exposed towards the outside (Fig. 4E). Because the exposure of the NAC region to water lowers the activation energy of intermolecular interactions, aggregation of αSyn could be triggered. Thus, we considered that the structural transition of monomeric αSyn, which was induced by Ca^2+^, promoted the aggregation of αSyn.

In addition, the representative structures of αSyn (8+) that were obtained using the gas-phase MD simulation (Supplementary Fig. 5) explained why Ca^2+^-bound αSyn tended to adopt a compact structure in the gas phase. From the various gas-phase structures of αSyn (8+), it was observed that the overall structures of the αSyn that were unbound and bound to Ca^2+^ (8+) were similar if their CCS_theo_ values were similar. To understand why compact conformation was preferred in Ca^2+^-bound αSyn, we investigated the representative compact structure of Ca^2+^-bound αSyn (CCS_theo_ = 1717.9 Å^2^) (Supplementary Fig. 6). The representative structure showed that the binding of Ca^2+^ to multiple carboxylate groups was preserved, and the electrostatic interaction between Ca^2+^ and the carbonyl backbone of residues in the N-terminal and NAC regions might be newly established during the ESI process (Supplementary Fig. 6). We predicted that this structural change was induced due to the absence of solvent molecules in the gas phase. Because electrostatic interaction becomes influential in the gas phase^50,51^, Ca^2+^, which had been bound only to C-terminal region, was additionally attracted to other regions of αSyn in the gas phase, thereby forming structures different from the solution structures.

Through the structural study of Ca^2+^-bound αSyn monomers, we observed that multiple Ca^2+^ ions bound to αSyn, and they could result in the formation of extended conformations of αSyn in solution (Fig. 4). We anticipated that the structural transitions of αSyn would be induced by the change in intramolecular interactions upon binding of Ca^2+^ to the C-terminal regions. In αSyn monomers, the N-terminal and C-terminal regions have long-range attractive interaction, because the N-terminal regions are positively charged and the C-terminal regions are negatively charged^12,13^. However, when Ca^2+^ ions were bound to the C-terminal regions, they would not attract the N-terminal regions. Thus, it was expected that the population of the compact structures reduced, which resulted in an increased average *R*_*g*_ value of Ca^2+^-bound αSyn.

From our results, it was considered that the structural changes in αSyn promoted its aggregation with the exposure of the NAC region. However, in addition to the structural aspect, charge neutralization of the C-terminal region may contribute to the induction of αSyn aggregation by reducing the repulsion between αSyn molecules, as suggested in the previous study of αSyn at low pH^52^. Therefore, it was considered that the aggregation of αSyn may be additionally accelerated by the change in local charge environment of the C-terminal region.

### Interfibrillar aggregation of αSyn induced by Ca^2+^

**Figure 5:**
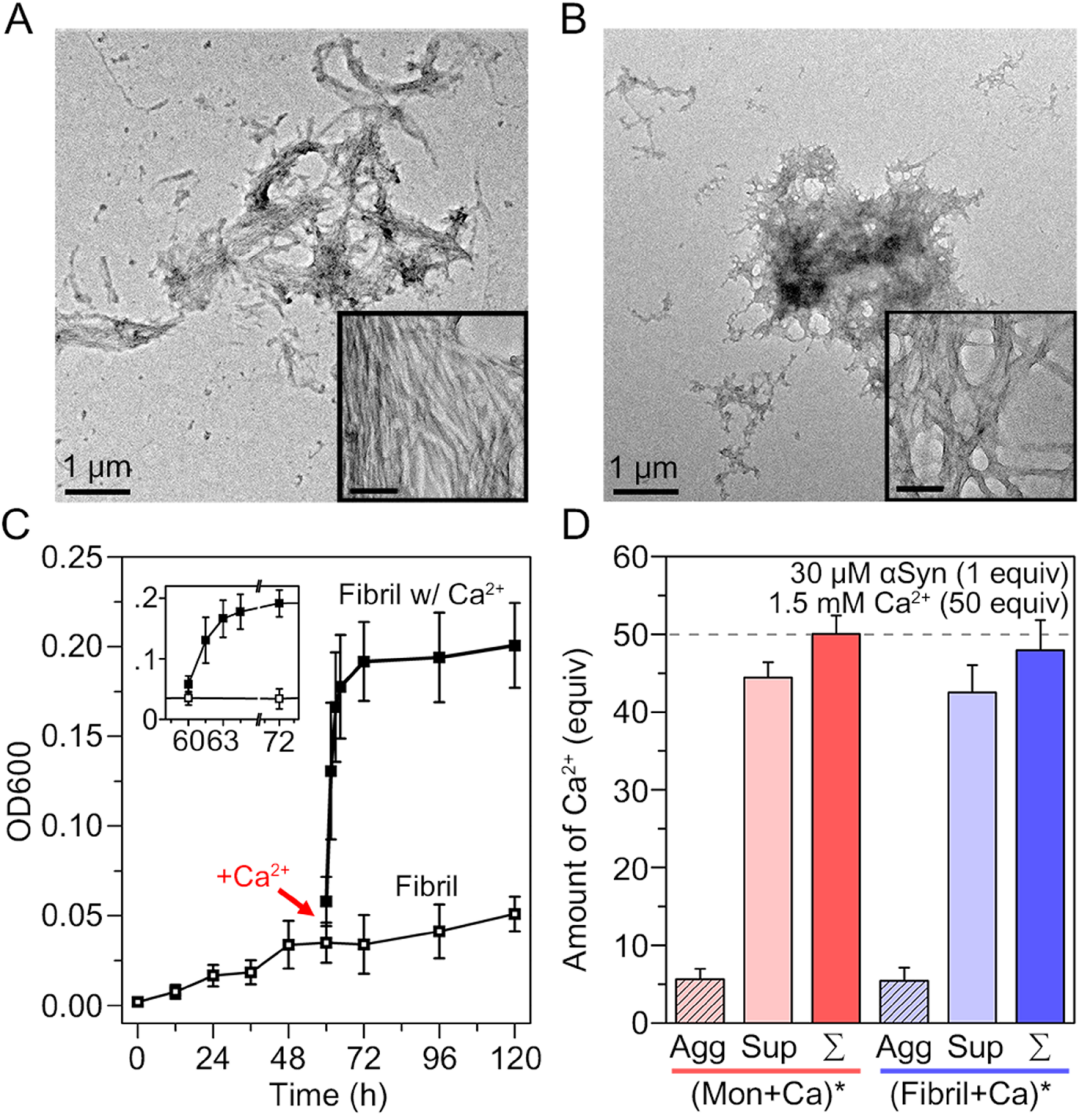
Ca^2+^-mediated interfibrillar aggregation of αSyn. TEM images of (**A**) αSyn aggregates formed by the addition of Ca^2+^ to mature αSyn fibrils (αSyn solution incubated for 60 h) and (**B**) aggregates formed by the addition of Ca^2+^ to αSyn, which was pre-incubated for 30 h in the absence of Ca^2+^ (~*t*_1/2_). For the experiments, 3.75 μL of 100 mM CaCl_2_ in 20 mM Tris-HCl was added to 246.25 μL of pre-incubated 30.46 μM αSyn to make solution with final concentrations of 30 μM αSyn and 1.5 mM Ca^2+^ (we assumed that the solution volume was conserved during the incubation). The scale bars in the insets of TEM images are 100 nm. (**C**) Measurement of OD600 of αSyn samples during fibril formation from monomers and interfibrillar aggregation induced by additionally added Ca^2+^. As a control, 30.46 μM αSyn was incubated without Ca^2+^. (**D**) Amounts of Ca^2+^ (50 equivalent in total, concentration of 1.5 mM) included in the αSyn aggregates (1 equivalent, concentration of 30 μM) formed through Ca^2+^ mediation. Agg, Sup, ∑ denote the amounts of Ca^2+^ in insoluble aggregates, supernatants, and the sum of the Ca^2+^ in aggregates and supernatants. ()* denotes the aggregates that were formed from the components enclosed within parentheses, monomers (Mon) or preformed fibrils with Ca^2+^.

Our TEM image of αSyn aggregates formed through Ca^2+^ mediation demonstrated that Ca^2+^ induced the formation of large αSyn aggregates through interfibrillar aggregation (Fig. 1D). In order to understand the role of Ca^2+^ in αSyn interfibrillar aggregation, we investigated whether Ca^2+^ can also induce interfibillar aggregation when it is added to mature αSyn fibrils. The mature αSyn fibrils were prepared by incubating αSyn monomer for 60 h, when the fibrillation extent was maximum (Fig. 1B). Surprisingly, we observed that the fibrils were converted to large aggregates, which were similar to the aggregates that were formed by the initial application of Ca^2+^ to the αSyn monomers (Fig. 5A). The inset in Fig. 5A obviously showed that the aggregates were formed through interfibrillar aggregation. We also observed that the aggregates had similar structural characteristics, such as low CD intensity and low structural portion of β-sheets as demonstrated in the IR spectrum, compared with the aggregates formed by initial Ca^2+^ addition (Supplementary Fig. 7).

To understand the properties of interfibrillar αSyn aggregation, we monitored the morphological changes upon the addition of Ca^2+^ to αSyn samples at different stages of fibrillation. As shown in Fig. 5B, large aggregates composed of fibrils were formed even when Ca^2+^ was added to the αSyn solution, which was incubated for 30 h (*t*_1/2_). In contrast, the αSyn samples that were incubated for 30 h and 60 h without Ca^2+^ showed the presence of conventional fibrils (Supplementary Fig. 8). OD measurements further showed the kinetics of Ca^2+^-mediated aggregation between fibrils (Fig. 5C). During the conversion of αSyn monomers to amyloid fibrils in the absence of Ca^2+^, the OD at 600 nm (OD600) increased gradually to approximately 0.05. However, when Ca^2+^ was added after 60 h of the incubation of αSyn, OD600 increased instantaneously, and reached approximately 0.20 in a few hours of incubation (Fig. 5C). This sharp increase in OD value indicated that the rates of Ca^2+^-mediated interfibrillar aggregation was faster than the aggregation kinetics of αSyn monomers incubated with Ca^2+^, which required ~20 h (Fig. 1B). This was possibly because the time required to form amyloid fibril was further included when monomers were incubated with Ca^2+^ in addition to the interfibrillar aggregation step. Moreover, we observed that interfibrillar aggregation subsequently commenced when fibrils started to form from αSyn incubated with Ca^2+^ (Supplementary Fig. 8). Therefore, it is suggested that Ca^2+^ promptly mediates interfibrillar aggregation at any time in the presence of αSyn fibrils.

All of the results indicate that Ca^2+^ directly mediates the aggregation between αSyn fibrils. However, it is not clear whether Ca^2+^ is incorporated in the aggregates. Therefore, we performed inductively coupled plasma optical emission spectroscopy (ICP-OES) to measure the amount of Ca^2+^. For the ICP-OES experiment, incubated αSyn samples were centrifuged at 18,000 × g, and the amounts of Ca^2+^ were measured in supernatant and insoluble αSyn aggregates. Figure 5D shows that the aggregates formed by incubating Ca^2+^ with αSyn monomer and fibril both include significant amount of Ca^2+^. Because Ca^2+^ enhanced the affinity between the C-terminal region of fibrils in both cases, it was considered that Ca^2+^ reduced the charge–charge repulsion between acidic residues of adjacent fibrils by binding to the acidic residues. In addition, we observed that the amounts of incorporated Ca^2+^ were similar in both aggregates (Fig. 5D). We expected that these similar amounts of Ca^2+^ may be due to the identical role of Ca^2+^ in both cases, at least with regard to interfibrillar aggregation.

### The aggregation mechanism of αSyn mediated by Ca^2+^

In the present study, we have shown that Ca^2+^ interacts with αSyn, and mediates distinct pathways of aggregation. At the early stage of aggregation, Ca^2+^, which binds to the C-terminal region of αSyn, induces structural transition of the protein monomer, whereby the NAC region is exposed, thereby resulting in rapid fibrillation. Then, Ca^2+^ triggers nonspecific interfibrillar aggregation to produce large aggregates as the final products.

**Figure 6:**
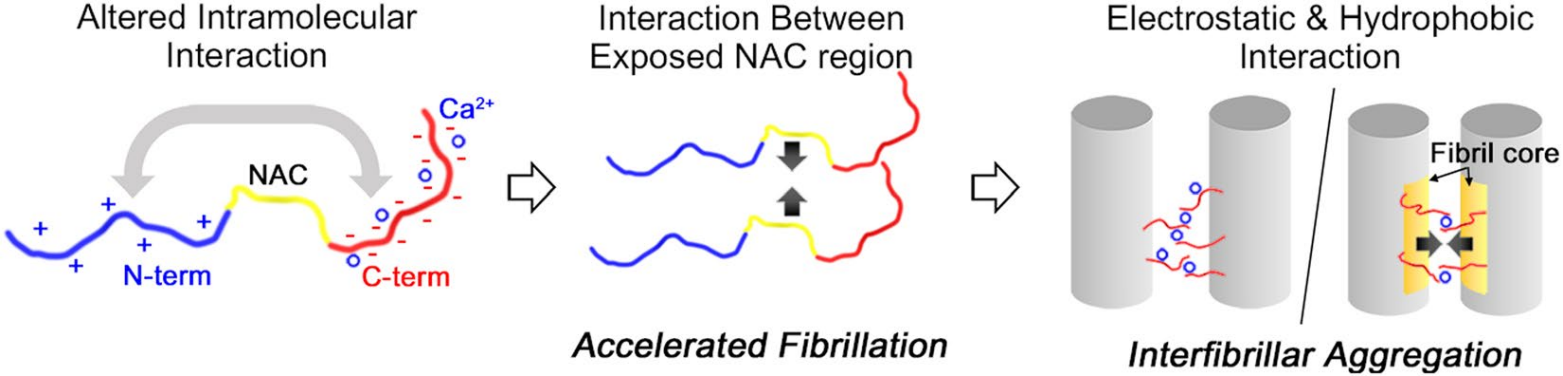
Mechanism of Ca^2+^-mediated aggregation of αSyn.

When Ca^2+^ ions are bound to αSyn monomers, the population of αSyn conformation is changed and fibrillation rate of αSyn is increased. Thus, the attractive intramolecular interactions between the N- and C-terminal regions were likely reduced due to the positive charge of Ca^2+^ ions bound to the C-terminal region of αSyn monomers. This change in intramolecular interaction decreases the stability of monomeric αSyn, by inducing the hydrophobic NAC region exposed to water. Therefore, the Ca^2+^-bound monomers begin to undergo fibrillation to prevent exposure of their hydrophobic regions to water (Fig. 6).

Using ThT assay, TEM, and CD spectroscopy, we found that the secondary structure of αSyn rapidly changed, becoming rich in β-sheets and forming large interfibrillar aggregates, in the presence of Ca^2+^. However, the secondary structures of the aggregates finally formed through Ca^2+^ mediation were different from those of the conventional fibrils (Fig. 3D). We examined whether this structural difference originated from the structure of fibril itself or formed during interfibrillar aggregation. Our results that were obtained upon the addition of Ca^2+^ to mature αSyn fibril provided a clue to resolve the issue. We observed that the secondary structures of the aggregates that were formed through Ca^2+^ mediation were similar regardless of whether Ca^2+^ was added to monomeric form or fibrillar form of αSyn (Supplementary Fig. 7). We considered that if the distinctive secondary structure of αSyn aggregates formed by initial addition of Ca^2+^ were merely a property of individual fibrils, the mature fibrils forming large aggregates by Ca^2+^ would have undergone structural change before the interfibrillar aggregation. However, based on the recently reported structure of αSyn fibril^53^, the binding of Ca^2+^ to a single strand of αSyn fibril would not be sufficient to alter the overall structure of fibril. The structure showed that the C-terminal region of αSyn fibril is located far from the N-terminal region, while the residues 30–100 in the middle form the fibril core^53^. This implied that the N-terminal region would not be affected by Ca^2+^-bound C-terminal region. Additionally, the structure showed that the fibril core region does not have a strong interaction with the C-terminal region. Thus, it was considered that the structural change of secondary structure of αSyn aggregates may occur during interfibrillar aggregation.

Semerdzhiev *et al*. recently reported that the enhanced ionic strength of the solution induces interfibrillar aggregation of αSyn^22^. They suggested that the aggregation occurs *via* long-ranged repulsive and short-ranged attractive interactions^22^. In our study, we demonstrated that the direct interaction between Ca^2+^ and αSyn fibrils causes interfibrillar aggregation of αSyn. The OD measurements showed that the interfibrillar aggregation of preformed αSyn fibrils is immediately initiated after Ca^2+^ was added (Fig. 5C). Since Ca^2+^ mainly binds to acidic residues in the C-terminal region and remain in the finally formed aggregates (Fig. 5D), (1) the incorporated Ca^2+^ may reduce the interfibrillar repulsion between negatively charged C-terminal residues, and (2) the interfibrillar interaction may be further stabilized through the chelating Ca^2+^ between two acidic residues (*i.e*. originated from each fibril). In addition, our IR spectra showed the dramatic decrease in β-sheet abundance of Ca^2+^-mediated fibrils (Fig. 3D and Supplementary Fig. 7B). However, changing the secondary structure cannot be explained with the only electrostatic interaction between Ca^2+^ and C-terminal regions, because β-sheet structure mostly originates from the fibril core region^53^. β-sheet is one of the most stable secondary structures and the fibril core region is composed of a large number of hydrophobic residues. Therefore, the structural change in this region implies that hydrophobic interaction was newly formed between the fibril core regions of different fibrils. Based on the structural analysis, we characterized the aggregation mechanism of αSyn mediated by Ca^2+^ (Fig. 6); at first, the fibrils are closely located due to the electrostatic interaction formed between divalent metal ions and C-terminal regions of fibrils; then, the hydrophobic core regions of adjacent fibrils are aggregated with a partial reorientation in the core structures of fibrils.

### Cytotoxicity of αSyn fibrils and Ca^2+^-mediated aggregates

**Figure 7:**
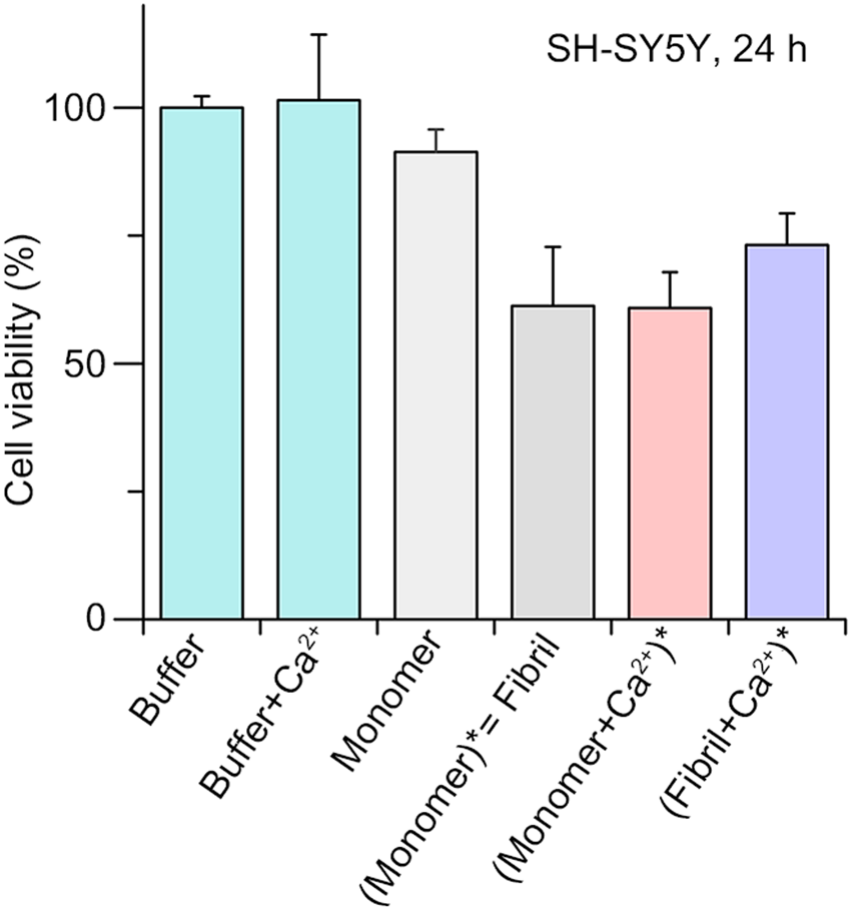
Results of the cell viability assay using SH-SY5Y cells after incubation for 24 h with each αSyn sample. ()* denotes the aggregates that were formed from the components enclosed within parentheses.

The fact that aggregation of both αSyn monomer and fibril are influenced by high level of Ca^2+^, which is similar to that of extracellular fluid, indicates that the observed Ca^2+^-mediated αSyn aggregation could be induced in the cells undergoing dysregulation of Ca^2+^ homeostasis or in the Ca^2+^-rich extracellular space. Because of the relationship between dysregulated Ca^2+^ homeostasis and α-synucleinopathies, we investigated whether aggregates formed through Ca^2+^ mediation have cytotoxicity, using methylthiazolyldiphenyl-tetrazolium bromide (MTT) assay with SH-SY5Y neuroblastoma cells. Compared to the control groups (buffers and monomer), the αSyn aggregates, which were formed by the initial addition of Ca^2+^ were as toxic as normal αSyn fibrils (cell viability of 61%), while the aggregates that were formed through Ca^2+^ mediation from preformed fibrils had slightly reduced cytotoxicity (cell viability of 73%) (Fig. 7). Our results showed that both Ca^2+^-mediated αSyn aggregates are cytotoxic regardless of when Ca^2+^ is added during the aggregation processes. This supports that the dysregulated Ca^2+^ homeostasis or secretion of αSyn to Ca^2+^-rich extracellular space is a potential pathogenesis of the diseases related to α-synucleinopathies.

## Conclusions

We have observed the formation of large interfibrillar aggregates of αSyn associated with hard divalent metal cations. Then, we characterized the pathway of αSyn aggregation mediated by Ca^2+^, which was a representative hard divalent metal ion, by using various biophysical techniques. Our results demonstrated that multiple Ca^2+^ ions bound to the C-terminal region of αSyn stimulates the structural transition of αSyn monomers that exposes the NAC region. This structural change accelerated αSyn fibrillation by lowering the activation energy for intermolecular interactions between the αSyn molecules. In addition, we observed that Ca^2+^ induced interfibrillar aggregation *via* electrostatic interaction between Ca^2+^ and the C-terminal regions, and hydrophobic interactions between the fibril core regions. Our cytotoxicity results suggested that the interaction between Ca^2+^ and αSyn accelerated the formation of toxic αSyn aggregates. As Ca^2+^ is the most abundant divalent metal ion in extracellular fluid (e.g., the synaptic cleft) and is a critical physiological factor for αSyn fibrillation among hard divalent ions, our results suggested the importance of the interaction between Ca^2+^ and αSyn in α-synucleinopathies. Furthermore, the detailed examination of the structures and the molecular interactions during αSyn aggregation would be valuable to understand the pathology of α-synucleinopathies.

### Data availability

All data generated or analysed during this study are included in this published article (and its Supplementary Information files).

## Electronic supplementary material

Supplementary Information
