# Supplementary material for: Molecular Role of Ca2+ and Hard Divalent Metal Cations on Accelerated Fibrillation and Interfibrillar Aggregation of α-Synuclein
Source: Sci Rep. 2018 Jan 30;8:1895. doi: 10.1038/s41598-018-20320-5 (PMC5789889; doi:10.1038/s41598-018-20320-5)
Supplement: Supplementary file 1 — Supplementary Information [file 41598_2018_20320_MOESM1_ESM.pdf]

Supplementary Information

Molecular Role of Ca^{2+} and Hard Divalent Metal Cations on Accelerated Fibrillation and Interfibrillar Aggregation of α-Synuclein

*Jong Yoon Han, Tae Su Choi, and Hugh I. Kim**

Department of Chemistry, Korea University, Seoul 02841, Republic of Korea

* To whom correspondence should be addressed: E-mail: hughkim@korea.ac.kr

Materials and Methods

Preparation of αSyn samples. Alpha-synuclein (αSyn), which was purchased from rPeptide (Bogart, GA, USA), was suspended at 1 mg/mL in water (the protein powder contains 20 mM Tris-HCl 100 mM NaCl, pH 7.4), and the buffer was exchanged to 20 mM Tris-HCl (pH 7.4) by centrifugation using a Amicon® 10K centrifugal filter. To remove possible preformed aggregates, the αSyn solution was centrifuged at 18,000 × g for 10 min, and the supernatant was taken from the solution. The concentration of the αSyn stock solution was directly estimated using the molar extinction coefficient, $\varepsilon_{280} = 5{,}960\ M^{-1}\ cm^{-1}$. For the preparation of the fibrils and the aggregates formed with Ca^{2+} mediation, 30 μM of freshly prepared αSyn, in the presence or absence of 1.5 mM calcium chloride ($CaCl_2$) (Sigma-Aldrich, St Louis, MO, USA) was added (total volume of 250 μL) to 0.3-mL borosilicate glass vials with a V-shaped bottom (Wheaton, USA, Millville, NJ, cat. no. 03-410-020). For all sample preparation, 1 M $CaCl_2$ stock in water was used, of which the concentration was determined through titration against ethylenediaminetetraacetic acid (EDTA) (Sigma-Aldrich, St Louis, MO, USA).[1] The samples in glass vial were incubated at 37 °C with agitation at 200 rpm.

For small-angle x-ray scattering (SAXS) experiment, rigorous separation was performed in order to obtain absolutely pure monomeric protein. Because the X-ray scattering intensity is proportional to the square of the molecular weight ($I(0) \propto N \cdot m^2$, where $I(0)$ is the extrapolated scattering intensity at an angle of zero, N is the number of particles, and m is the number of electrons of the particle),[2] even a trace amount of protein oligomers has significant influence on SAXS scattering profile of monomers. Therefore, the αSyn solution was filtered using a Amicon® 100K centrifugal filter (Millipore, Eschborn, Germany) to remove small oligomers that possibly coexisted.[3,4] Although αSyn has a low molecular weight (14 kDa), αSyn could not be filtered using

30K and 50K filters, possibly because αSyn is intrinsically disordered and has large R_g values of ~ 31.8 ± 0.7 Å (Fig. 4B) comparable to that of human serum albumin (66 kDa protein, R_g values of ~ 30.4 ± 0.5 Å).[5] After 100K filtration, the filtrate was concentrated using a 10K centrifugal filter, and used for the SAXS experiment. We confirmed that this additional step helped to obtain SAXS profiles with reduction of trace amounts of oligomers. However, no significant difference was observed in the aggregation kinetics between the 100K-filtered αSyn and unfiltered αSyn (Supplementary Fig. 9), indicating that the amount of αSyn oligomers included in the 100K-filtered samples was influential in the SAXS experiment but not sufficiently significant to affect the overall aggregation kinetics.

Thioflavin T (ThT) fluorescence assay. Thioflavin T (ThT) was purchased from Sigma-Aldrich (St Louis, MO, USA). The ThT solution was prepared at a concentration of 10 µM in 20 mM Tris-HCl (pH 7.4). At every 6 hours or 12 hours during incubation, the αSyn samples were dispersed by pipetting (5–10 times) and 3 µL aliquots were collected from the samples. Then, the 3 µL aliquots were mixed with 147 µL of ThT solution, and fluorescence was measured (ex: 446 nm, em: 482 nm) using a fluorescence spectrometer (FluoroMate FS-2, Scinco, Seoul, Korea). We measured the fluorescence intensity of 3 µL of Tris-HCl mixed with 147 µL of ThT solution, and subtracted the intensity as a baseline from that of the αSyn sample. After the measurement of the ThT fluorescence intensity, the data points obtained from each sample were fitted by using the following sigmoidal function (Eq. (1)).[6,7]

$$F(t) = \mathrm{r}_1 + \frac{\mathrm{r}_2 - \mathrm{r}_1}{1 + e^{-(t-t_0)/\tau}} \qquad (1)$$

Using the fitted function, the half time ($t_{1/2}$) was determined when the intensity of F(t) was equal to $\mathrm{r}_2/2$.

Transmission electron microscopy (TEM). αSyn solutions (30 μM) incubated with and without 1.5 mM Ca^{2+} for 7 days were dispersed by pipetting (5–10 times). Then, we took 5 μL of the suspension and mixed it with 5 μL water. 5 μL of the two-fold diluted sample was spotted on a TEM grid for 3 min, and removed. Then, 5 μL of 0.5% uranyl acetate solution was spotted for 1 min, and removed immediately. The TEM grids were dried under ambient conditions. The analysis was performed using a field-emission transmission electron microscope (Tecnai G2 F30ST, FEI co., USA) at the Korea Basic Science Institute (KBSI) in Seoul, Republic of Korea.

Circular dichroism (CD) spectroscopy. 30 μM αSyn samples in 20 mM Tris-HCl buffer with and without 1.5 mM $CaCl_2$ were incubated at 37 °C with agitation at 200 rpm for 0 h, 12 h, 24 h, and 7 d. At each time point, the incubated sample was fully dispersed by pipetting and 100 μL of the sample was collected from the vial. The sample was diluted two-fold by using 20 mM Tris-HCl. Then, the two-fold diluted αSyn samples were dispersed again and immediately inserted into a cuvette with a path length of 0.1 cm (Starna, Atascadero, CA, USA), and CD was measured on a J-1100 CD spectrometer (Jasco, Tokyo, Japan). The spectra were obtained from 195 to 250 nm at a scan speed of 50 nm/min. All spectra were obtained by averaging spectra of five replicate scans.

Infrared (IR) spectroscopy. All incubated αSyn samples (30 μM αSyn with and without 1.5 mM $CaCl_2$) were concentrated using 100K centrifugal filters before subjecting to IR spectroscopy. The monomeric αSyn sample was concentrated using 10K centrifugal filter. The concentrated αSyn samples were dropped onto calcium fluoride windows, and dried at 37 °C. Then, the samples were analyzed using a Bruker Alpha Fourier transform infrared (FTIR) spectrometer (Bruker Optik GmbH, Ettlingen, Germany) with a resolution of 4 cm^{-1}. For all IR absorbance spectra, the linear baseline between the spectrum point of 1700 and 1600 cm^{-1} was subtracted from the spectra, and

the spectra were normalized to an identical area between the spectra and baseline within 1600~1700 cm^{-1}.

Small-angle X-ray scattering (SAXS). The SAXS experiments were performed at 4C SAXS II beamline of the Pohang Accelerator Laboratory (PAL). The sample-to-detector distance was set to 2 m, and all experiments were performed at 20 °C. αSyn solution (>300 μM) in 20 mM Tris-HCl buffer (pH 7.4) was freshly prepared following the sample preparation method described above, and placed at a temperature of 4 °C. The αSyn solution was used within 30 minutes for SAXS experiments because αSyn started to form aggregates after longer storage times. For the measurements, 200 μM αSyn solution (2.89 mg/mL) in the presence and absence of $CaCl_2$ was prepared. Scattering profiles were recorded six times for 5 s, and averaged. The values of the radius of gyration (R_g) of αSyn were estimated using the Guinier relationship.[2] All the scattering profiles that were used for the analysis were confirmed not to include aggregates in the solution using the Eq. (2).[8]

$$MW_p = \frac{I(0)_p}{c_p} \frac{MW_{st}}{I(0)_{st}/c_{st}} \qquad (2)$$

where $I(0)$ is the extrapolated scattering intensity at zero angle, c is the protein concentration in mg/ml, and *p* and *st* denote protein and standard, respectively. Using Ca^{2+}-free αSyn as the standard, the molecular weight (MW) of αSyn in the presence of Ca^{2+} could be estimated. The similar values of $I(0)$ of Ca^{2+}-free αSyn and αSyn with different Ca^{2+} concentrations indicated that neither was more aggregated than the other (Supplementary Fig. 3)

Generation of random αSyn pool. At pH 7.4, αSyn is likely to be present in the −9 charge state. For the generation of the −9 charge state, all lysine (Lys) residues and N-terminus were protonated, and all glutamic acid (Glu), aspartic acid (Asp) residues, and C-terminus were deprotonated. A

previously reported structure of micelle-bound αSyn (pdb code 1XQ8) was used for molecular dynamics (MD) simulation using GROMACS 4.5.5.[9] αSyn was modeled using the AMBER-99SB-ILDN force field,[10] and LINCS[11] was used for constraining all bonds. To remove the abundant helical structures in micelle-bound αSyn (1XQ8), MD simulation was performed for 1 ns at 460 K using a generalized Born implicit solvent model.[12] After MD simulation for 1 ns, it was confirmed that most of the helical structures of αSyn became random coil structures. For the efficient modeling of αSyn structures in solution using the prepared initial αSyn structure, replica-exchange molecular dynamics (REMD) simulation was performed.[13] For REMD simulation, a generalized Born implicit solvent model and five replicas at 430, 445, 460, 470, and 490 K were used (average exchange probability of ~ 0.15). The systems were independently equilibrated at each temperature for 1 ns, and then, subjected to REMD simulations with exchange attempted at intervals of 2 ps. Each replica was simulated for 20 ns, and hence, the total simulation time was 100 ns. Then, 2,000 structures were extracted at each temperature (10,000 in total), and all the structures collectively served as a random pool to search for structures in solution.

Ensemble optimization method (EOM). Ten thousand αSyn models were converted into theoretical SAXS profiles, and the best ensembles that matched the experimental profiles were sought using the program GAJOE[14,15] in the program suite ATSAS 2.7.0.[16] Fifty ensembles, fifty curves per ensemble (fixed), and one hundred genetic algorithm repetitions were used for our EOM analysis. For the optimization of an ensemble using experimental αSyn profiles in the presence of Ca^{2+}, the same αSyn REMD pool was used, and it was confirmed that the theoretical SAXS profiles were not changed after the modeling of binding of four Ca^{2+} ions to the structures (Supplementary Fig. 10). For the generation of Ca^{2+}-bound structures, four Ca^{2+} ions were added near the amino acid residues 114-115 (ED), 119-123 (DPDNE), 130-131 (EE), and 137-139 (EPE). Then, energy

minimization was performed with distance restraint between Ca^{2+} and two proximate oxygen atoms in the carboxylate ion using GROMACS 4.5.5.[9] The distance restraint was described using piecewise harmonic potential with a force constant of k=50 kJ mol^{-1} nm^{-1} at the distance lower than 2.0 Å and higher than 2.5 Å. The potential was set zero at the distance between 2.0 Å and 2.5 Å.

Gas-phase MD simulation and calculation of theoretical collision cross-section (CCS) values. The 50 structures that were obtained from EOM were used as the initial structures for gas-phase MD simulation. For generation of the +8 charged αSyn, all Lys residues and N-terminus were protonated, and C-terminus were deprotonated. Among Glu and Asp residues, 7 residues were selected and deprotonated. Then, MD simulation *in vacuo* at 300 K was performed for 20 ns using the 50 initial structures in solution. However, this charge assignment method does not generate the extended conformation of which theoretical CCS values (CCS_{theo}) > 2500. Instead, the structures of which CCS_{theo} > 2500 in Supplementary Fig. 5, were generated using deprotonation of 8 acidic residues (Glu and Asp) and protonation of C-terminus. For the generation of the +8 charge state of four-Ca^{2+}-bound αSyn molecules, similarly, all Lys residues and N-terminus were protonated with two options: 1) deprotonation of C-terminus and 15 acidic residues, 2) protonation of C-terminus and deprotonation of 16 acidic residues. In order to describe binding of Ca^{2+} on the region 114-115 (ED), 119-123 (DPDNE), 130-131 (EE), and 137-139 (EPE), the neighboring two acidic residues per Ca^{2+} were deprotonated. MD simulation *in vacuo* was also performed in the same manner. CCS_{theo} values of the final structures were calculated using the exact hard sphere scattering (EHSS) method in MOBCAL.[17]

Electrospray ionization-ion-mobility-mass spectrometry (ESI-IM-MS). ESI-IM-MS was performed using a Waters Synapt G2 HDMS quadrupole traveling wave ion mobility (TWIM)

orthogonal time-of-flight mass spectrometer (Waters, Manchester, UK). 20 μM αSyn solutions in 20 mM ammonium acetate (pH 7.4) with increasing $CaCl_2$ were prepared for the ESI-IM-MS experiments. The sample was introduced into an Au/Pd coated borosilicate emitter (Thermo), and sprayed into the nano-ESI source at flow rate of 0.2 μL/min. The experiments were conducted at nano-ESI capillary voltage of 1.3 kV, cone voltage of 20 V, and source temperature of 50 °C. Helium cell gas flow and IMS cell gas (N_2) flow were 150 mL/min and 60 mL/min, respectively. TWIM wave velocity and wave height were set to 500 m/s and 24 V, respectively. The experimental ion arrival times were converted into collision cross-section values by calibrating using denatured ubiquitin, cytochrome c, and apo-myoglobin whose collision cross-section values were previously reported.[18,19] To confirm binding of multiple Ca^{2+} ions to αSyn, ESI-MS was performed again using another mass spectrometer, Agilent 6560 DTIMS-Q-TOF (Agilent Technologies, Santa Clara, CA, USA). The αSyn solution was injected into the mass spectrometer at a flow rate of 3 μL/min. The source temperature was 60 °C; sheath gas temperature was 60 °C; capillary voltage was 1.5 kV. Instead of N_2, He gas was introduced into the drift tube, and three radio frequency (RF) voltages (high-pressure funnel RF, trap funnel RF, and rear Funnel RF) were set to 100 V.

Optical density measurements. The incubated αSyn samples (30 μM) were transferred into a cuvette (Starna, CA, USA, cat. no. 16.40FQ-10), and the optical density at 600 nm (OD600) was measured using a Shimadzu UV-1800 spectrophotometer (Shimadzu, Kyoto, Japan).

Inductively coupled plasma-optical emission spectroscopy (ICP-OES). First, the residual Ca^{2+} concentration in 20 mM Tris-HCl was determined by using the standard addition method with ICP-OES. For the experiment, 20 mM Tris-HCl with different amounts of additional $CaCl_2$ (0–1.6 mM) were prepared using 1 M Tris-HCl, HPLC water, and 1 M $CaCl_2$ (of which the concentration

was determined through EDTA titration[1]). 40 μL of each solution with different amounts of Ca^{2+} were diluted with 960 μL of 20 mM Tris-HCl and 2 mL of HPLC water. Then, the intensity of Ca^{2+} emission line at 396.847 nm was measured by using ICP-OES (Agilent 730, Agilent Technologies, USA). Based on the linear correlation between the emission intensity and known added Ca^{2+} amount, and the assumption of trivial amount of Ca^{2+} in HPLC water, the residual Ca^{2+} concentration in 20 mM Tris-HCl buffer was determined (Supplementary Fig. 11A). In the same manner, the residual Ca^{2+} in 20 mM ammonium acetate and αSyn (30 μM)-dissolved Tris-HCl and ammonium acetate were determined (Supplementary Fig. 11B, C). Tris-HCl and ammonium acetate included 2.3 ± 0.5 and 2.5 ± 0.1 μM Ca^{2+} and αSyn (30 μM)-dissolved Tris-HCl and ammonium acetate included 14.0 ± 2.7 and 41.3 ± 2.1 μM Ca^{2+}, respectively (Supplementary Fig. 11C).

The linear relation function of the Ca^{2+} emission intensity of Tris-HCl with a different Ca^{2+} amount in Supplementary Fig. 11A was also used as a calibration curve to measure Ca^{2+} in the unknown samples, after the replacement of the values of the x-axis (the concentration increased by residual Ca^{2+}) with the actual concentrations. For the quantitation of Ca^{2+} incorporated in the αSyn aggregates formed through Ca^{2+} mediation (Fig. 5D), the sample was prepared by the following procedure. The αSyn samples (40 μL) were collected and centrifuged at 18,000 × *g* for 20 min. After centrifugation, white pellets of αSyn aggregates were observed on the bottom of tube. Then, the whole supernatant solution (40 μL) was aliquoted from the tube (tube #1). The remaining αSyn aggregates on the tube bottom were dispersed using another 40 μL of 20 mM Tris-HCl buffer (tube #2). Then, to each separate samples (tube #1 and #2), 10 μL of 100 mM EDTA and 50 μL of 16 M urea (Shinyo Chemical Co., Ltd, South Korea) were added (total volume of 100 μL). To denature αSyn aggregates into monomers and extract Ca^{2+} from the aggregates, the

solutions were incubated at 95 °C for 20 min. Then, 900 μL of 20 mM Tris-HCl buffer was added, and filtered by using a 0.2 μm Whatman Puradisc syringe filter (Whatman International, Maidstone, Kent, UK, cat. no. 6784-1302). The filtration was performed to exclude the possibility of instrumental damage by dust or insoluble particles. By using the optical density measurements, we confirmed that all αSyn aggregates were disaggregated under high concentration of urea (~8 M) and heat-induced denaturation (~95 °C). Then the filtered solutions were finally diluted to 3 mL with HPLC water and their Ca^{2+} emission intensities were measured. The intensities were converted to a concentration by using a calibration curve. For accurate measurement, we used the calibration curve that was obtained immediately prior to the sample analysis.

Cell culture and MTT cytotoxic assay. Human neuroblastoma cells (SH-SY5Y) were used for the cell toxicity assay. SH-SY5Y cells were grown in 1:1 mixture of Dulbecco's modified Eagle medium (DMEM) and Ham's F-12 medium (Welgene, Daegu, South Korea) supplemented with antibiotics and 10% fetal bovine serum, at 37 °C with 5% CO_2. For the cytotoxicity assay, cells were plated in 96-well polystyrene plates with 2×10^4 cells per well (100 μL of medium). After incubation for 24 h at 37 °C, the medium was removed, and 100 μL of 6-fold diluted αSyn samples in fresh medium ([αSyn] = 5 μM) was added to each well. The plates were then incubated for an additional 24 h at 37 °C. Cell viability was determined using the methylthiazolyldiphenyl-tetrazolium bromide (MTT) toxicity assay with the addition of 10 μL of 5 mg/mL MTT (Sigma-Aldrich, St Louis, MO, USA), which was dissolved in phosphate buffered saline (PBS), to each well. After incubation for 3 h at 37 °C, the solution was removed, and 100 μL of dimethyl sulfoxide (DMSO) was added to each well. Then, the plate was gently agitated for 30 min in the dark to dissolve purple precipitates. The absorbance of each well was measured at 540 nm using a SpectraMax i3x microplate reader (Molecular Devices, Sunnyvale, CA, USA). Averages from 3

replicate wells were used for each sample and control. Cell viability was calculated by dividing the absorbance of the wells with that of the wells of Tris-HCl buffer-treated cells (control).

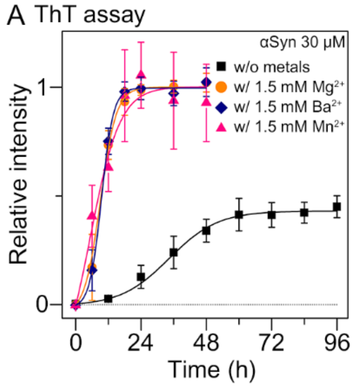

Supplementary Figure 1. ThT assay for the fibrillation kinetics of αSyn (30 μM) in the presence of 1.5 mM Mg^{2+}, Ba^{2+}, and Mn^{2+}. For the sample preparation, concentrations of metal ions were determined by using an EDTA titration method[1].

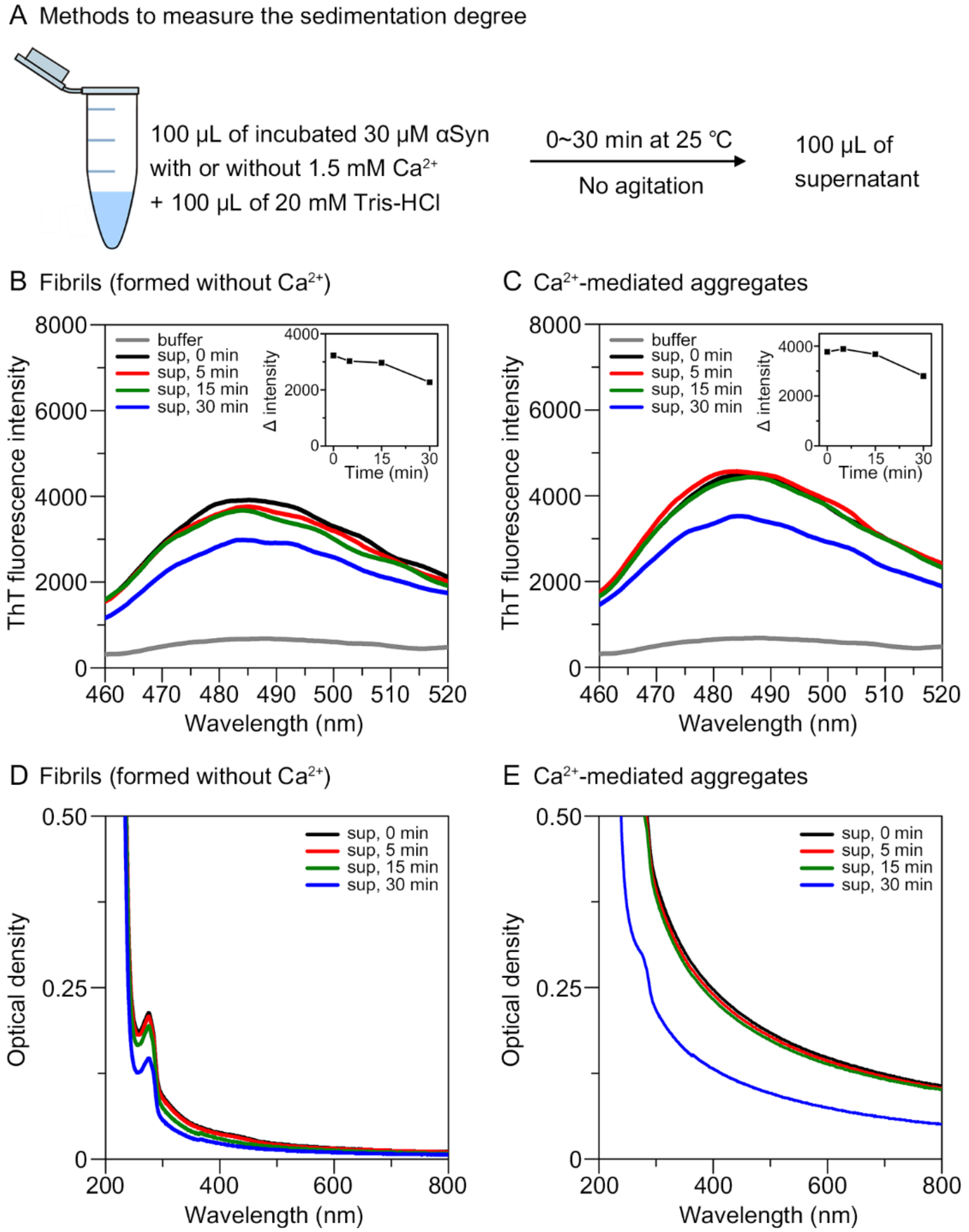

Supplementary Figure 2. Measurement of sedimentation degree of aggregates. A) Schematic diagram of the method used to measure the sedimentation of αSyn aggregates. The ThT fluorescence assay results obtained using the supernatant of B) fibrils and C) Ca^{2+}-mediated aggregates. Optical density of the supernatant of D) fibrils and E) Ca^{2+}-mediated aggregates.

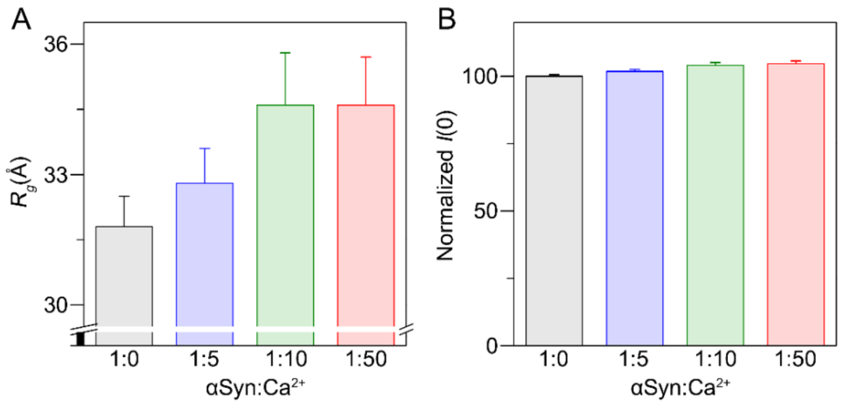

Supplementary Figure 3. SAXS results at various molar ratios of αSyn (200 μM) to Ca^{2+}. A) Radius of gyration (R_g) obtained using the Guinier analysis of SAXS scattering curves. B) Normalized $I(0)$ values of the scattering curves used for the analysis. The $I(0)$ value of αSyn:Ca^{2+}=1:0 was set as the standards for the comparison.

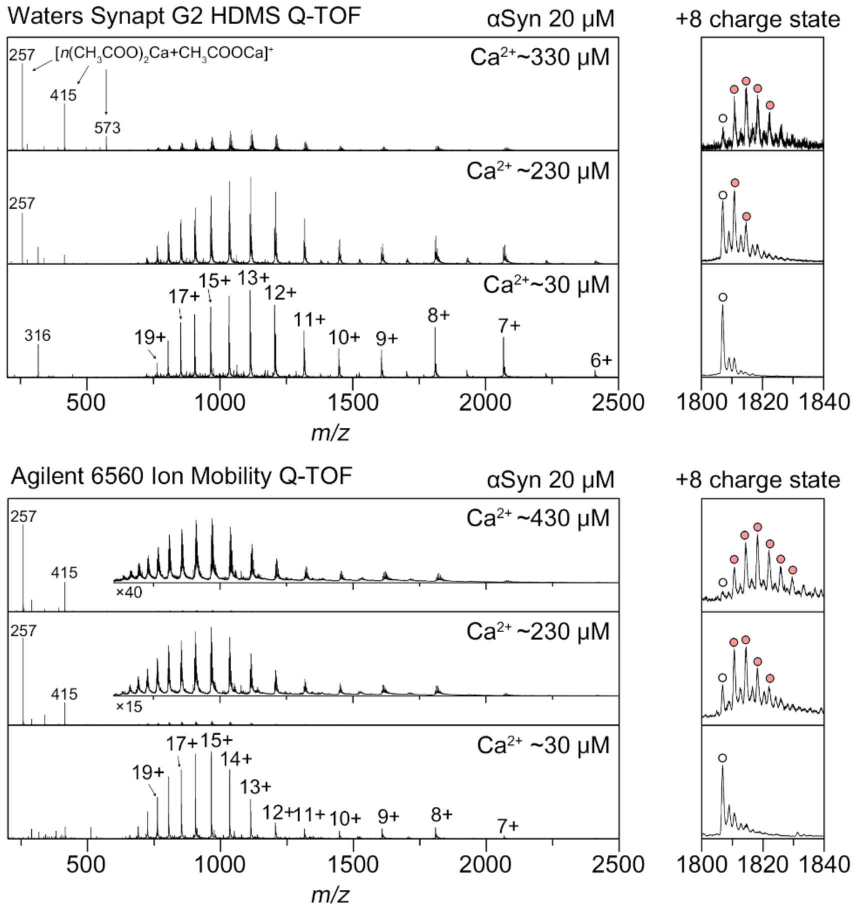

Supplementary Figure 4. ESI-MS spectra of αSyn (20 μM) with increasing Ca^{2+} concentrations. The spectra were obtained using Waters Synapt G2 HDMS Q-TOF and Agilent 6560 ion mobility Q-TOF mass spectrometers. The αSyn in the +8 charge state was shown as a representative image. The Ca^{2+}-unbound and bound αSyn peaks were marked with empty circles and filled circles, respectively. The peaks at *m/z* of 257, 415, and 573 correspond to calcium acetate cluster ions. With increasing Ca^{2+} concentration, the relative intensities of calcium acetate cluster ions increased and those of αSyn ions decreased in both mass spectrometers. The relative intensities of Ca^{2+}-unbound and bound αSyn ions were lowered by a $[Ca^{2+}]$ of 330 μM (Waters Synapt G2 HDMS) or 230 μM (Agilent 6560) due to the high concentration of Ca^{2+}, which implied that the total ion concentrations were above the charge saturation concentrations for our ESI in our experimental conditions.[20]

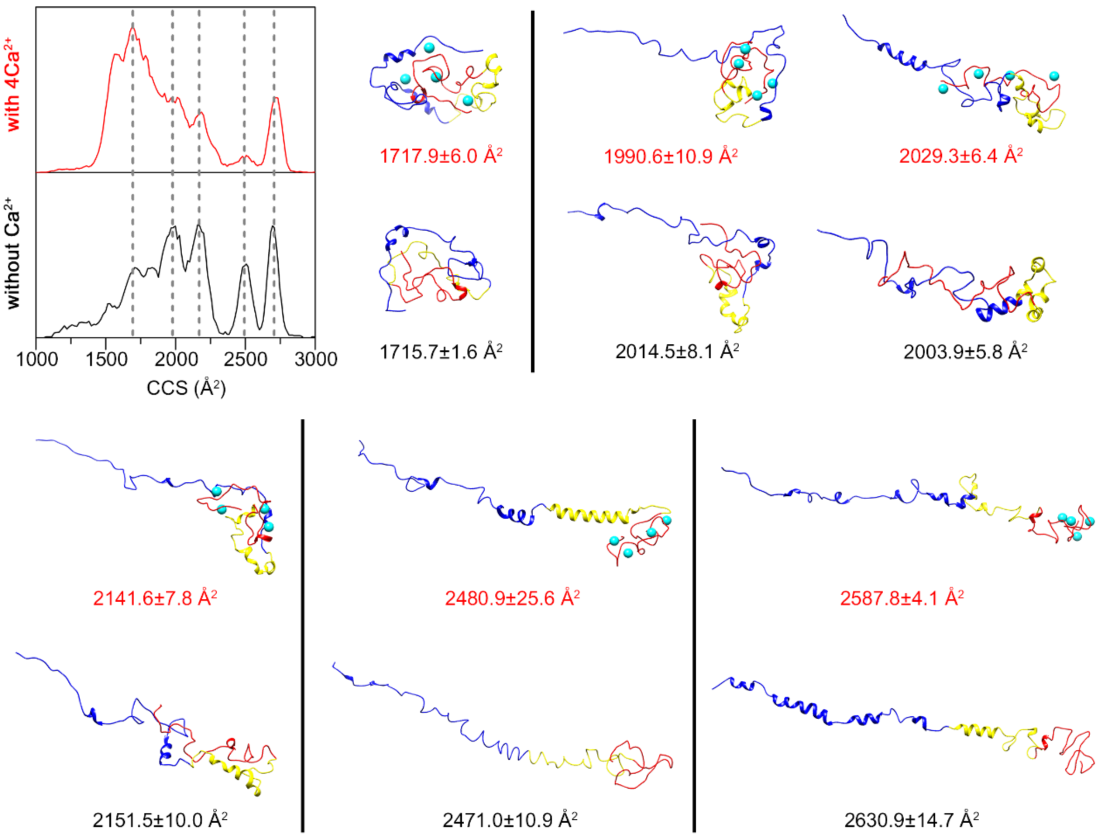

Supplementary Figure 5. Gas-phase structures of αSyn (+8) and theoretical collision cross-section values for each structure. Residues in blue, yellow, and red indicate the N-terminal, non-amyloid-β component (NAC), and C-terminal regions, respectively. Ca^{2+} ions are in cyan.

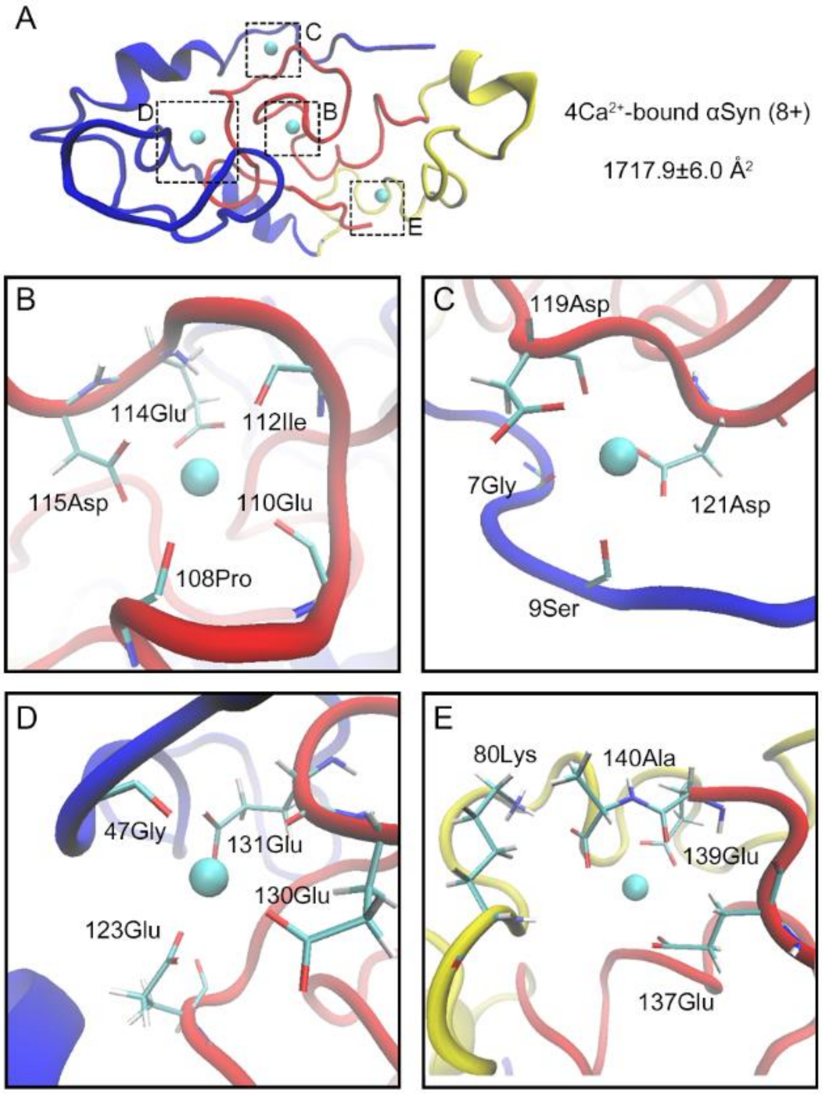

Supplementary Figure 6. Detailed gas-phase structure of 4 Ca^{2+}-bound αSyn (+8). Residues in blue, yellow, and red indicate the N-terminal, NAC, and C-terminal regions, respectively. Ca^{2+} ions are in cyan. Residues whose backbones play important roles in Ca^{2+} binding are illustrated with only their backbones.

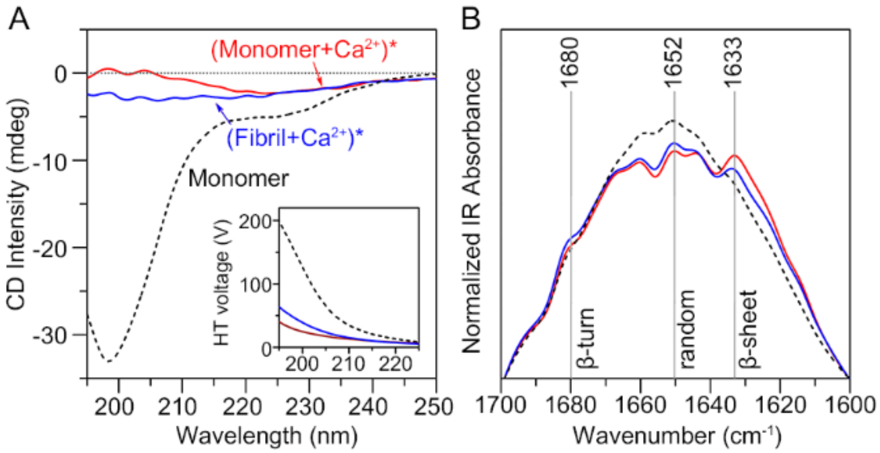

Supplementary Figure 7. Analysis of the secondary structures of αSyn aggregates, which were formed by the incubation of mature αSyn fibrils with Ca^{2+}. ()* denotes the aggregates that were formed from the components enclosed within parentheses. A) CD spectra. B) IR spectra.

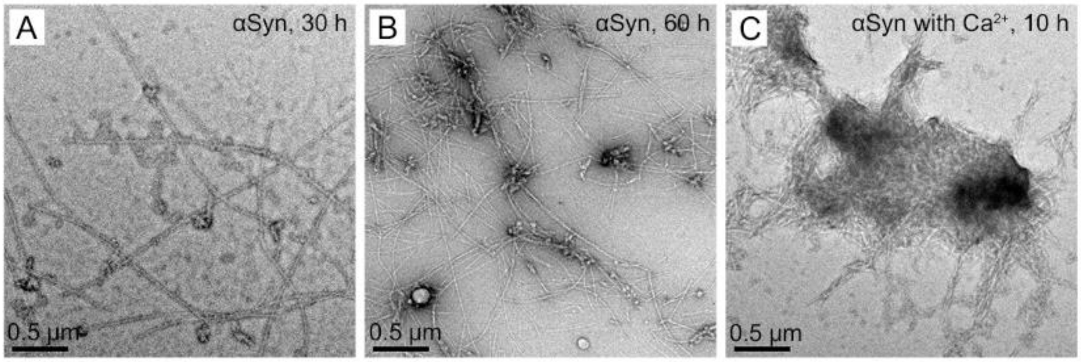

Supplementary Figure 8. TEM images of fibrils formed after A) 30 h and B) 60 h of the incubation of αSyn without Ca^{2+}. C) TEM image of the aggregates formed after 10 h of the incubation of αSyn with Ca^{2+}.

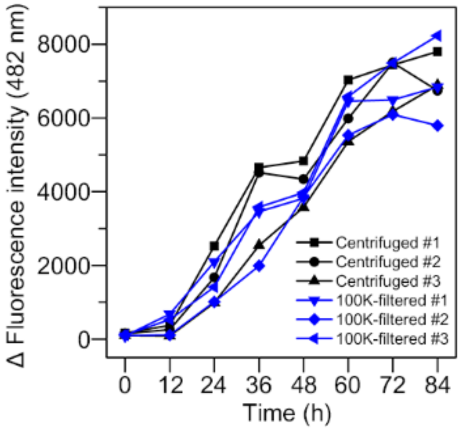

Supplementary Figure 9. ThT assay results of αSyn (30 μM) prepared following different sample preparation methods. Data points colored in black indicates αSyn samples prepared from supernatant after centrifugation at 18,000×g for 10 min. Data points colored in blue indicates αSyn samples prepared from solution filtrated through 100K filter.

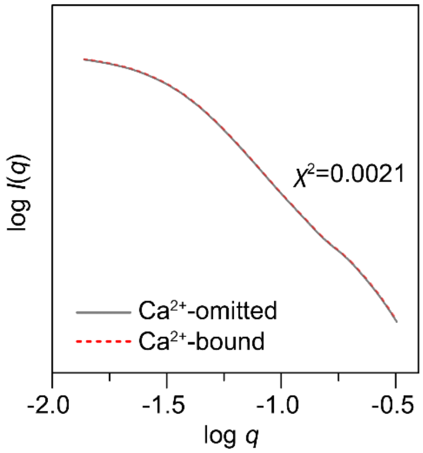

Supplementary Figure 10. Theoretical SAXS scattering profiles of αSyn ensemble (50 different structures) obtained from the EOM analysis of SAXS scattering profiles of αSyn with 50-fold Ca^{2+}, before and after the addition of four Ca^{2+} ions to the αSyn structures, and subsequent geometry optimization.

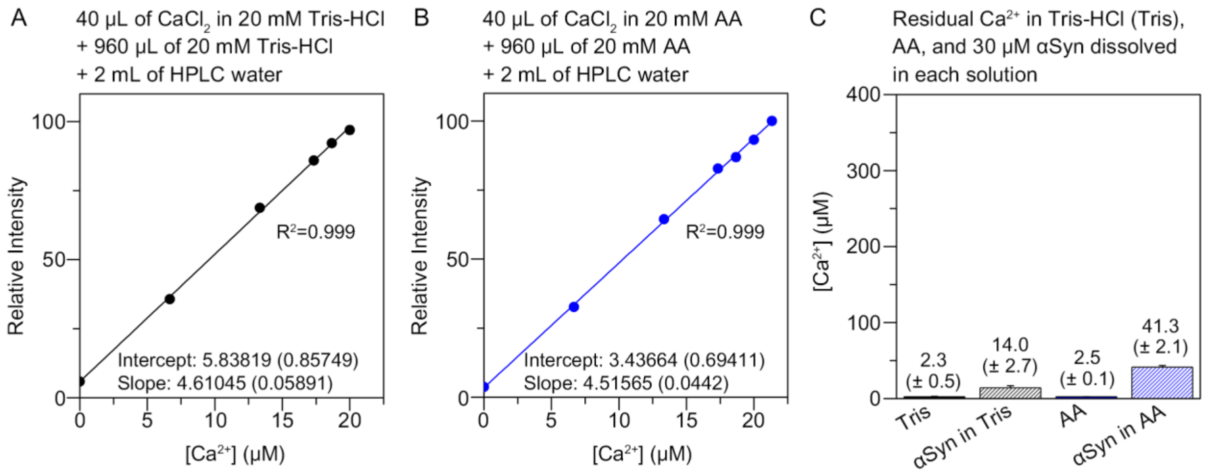

Supplementary Figure 11. Measurement of Ca^{2+} concentration by using ICP-OES. A) The measurement of residual Ca^{2+} in 20 mM Tris-HCl buffer by using the standard addition method. 40 µL aliquots of 0.0–1.6 mM $CaCl_2$ in 20 mM Tris buffer were diluted with 960 µL of 20 mM Tris-HCl and 2 mL HPLC water. On the assumption that HPLC water included an insignificant amount of Ca^{2+}, the residual Ca^{2+} concentration in 20 mM Tris buffer was determined (as shown in panel C). Considering the residual Ca^{2+} in Tris buffer, the corrected linear fitted function was used to determine the Ca^{2+} concentration in Fig. 5D. B) The measurement of residual Ca^{2+} determined in 20 mM ammonium acetate using the standard addition method. C) The determined residual Ca^{2+} concentration in 20 mM Tris-HCl (Tris), 20 mM ammonium acetate (AA), and each solution, including 30 µM αSyn.

References

1. Frank, J.W. The analytical uses of ethylenediamine tetraacetic acid. *D. Van Nostrand Co. Inc. Princeton, New Jersey* (1958).
2. Putnam, C.D., Hammel, M., Hura, G.L. & Tainer, J.A. X-ray solution scattering (SAXS) combined with crystallography and computation: defining accurate macromolecular structures, conformations and assemblies in solution. *Q. Rev. Biophys.* **40**, 191–285 (2007).
3. Khalaf, O. et al. The H50Q mutation enhances α-synuclein aggregation, secretion, and toxicity. *J. Biol. Chem.* **289**, 21856–21876 (2014).
4. Villar-Piqué, A. et al. Environmental and genetic factors support the dissociation between α-synuclein aggregation and toxicity. *Proc. Nat. Acad. Sci. USA* **113**, E6506–E6515 (2016).
5. Choi, T.S., Lee, H.J., Han, J.Y., Lim, M.H. & Kim, H.I. Molecular insights into human serum albumin as a receptor of amyloid-β in the extracellular region. *J. Am. Chem. Soc.* (2017).
6. Nielsen, L., Frokjaer, S., Brange, J., Uversky, V.N. & Fink, A.L. Probing the mechanism of insulin fibril formation with insulin mutants. *Biochemistry* **40**, 8397–8409 (2001).
7. Lee, C.-C., Nayak, A., Sethuraman, A., Belfort, G. & McRae, G.J. A three-stage kinetic model of amyloid fibrillation. *Biophys. J.* **92**, 3448–3458 (2007).
8. Mylonas, E. & Svergun, D.I. Accuracy of molecular mass determination of proteins in solution by small-angle X-ray scattering. *J. Appl. Cryst* **40**, s245–s249 (2007).
9. Hess, B., Kutzner, C., Van Der Spoel, D. & Lindahl, E. GROMACS 4: algorithms for highly efficient, load-balanced, and scalable molecular simulation. *J. Chem. Theory Comput.* **4**, 435–447 (2008).
10. Lindorff-Larsen, K. et al. Improved side-chain torsion potentials for the Amber ff99SB protein force field. *Proteins* **78**, 1950–1958 (2010).
11. MacKerell Jr, A.D. et al. All-atom empirical potential for molecular modeling and dynamics studies of proteins. *J. Phys. Chem. B* **102**, 3586–3616 (1998).
12. Onufriev, A., Bashford, D. & Case, D.A. Exploring protein native states and large-scale conformational changes with a modified generalized born model. *Proteins* **55**, 383–394 (2004).
13. Sugita, Y. & Okamoto, Y. Replica-exchange molecular dynamics method for protein folding. *Chem. Phys. Lett.* **314**, 141–151 (1999).
14. Bernadó, P., Mylonas, E., Petoukhov, M.V., Blackledge, M. & Svergun, D.I. Structural characterization of flexible proteins using small-angle X-ray scattering. *J. Am. Chem. Soc.* **129**, 5656–5664 (2007).
15. Tria, G., Mertens, H.D., Kachala, M. & Svergun, D.I. Advanced ensemble modelling of flexible macromolecules using X-ray solution scattering. *IUCrJ* **2**, 207–217 (2015).
16. Petoukhov, M.V. et al. New developments in the ATSAS program package for small-angle scattering data analysis. *J. Appl. Cryst* **45**, 342–350 (2012).
17. Mesleh, M., Hunter, J., Shvartsburg, A., Schatz, G.C. & Jarrold, M. Structural information from ion mobility measurements: effects of the long-range potential. *J. Phys. Chem.* **100**, 16082–16086 (1996).
18. Ruotolo, B.T., Benesch, J.L., Sandercock, A.M., Hyung, S.-J. & Robinson, C.V. Ion mobility–mass spectrometry analysis of large protein complexes. *Nat. Protoc.* **3**, 1139–1152 (2008).
19. Bush, M.F. et al. Collision cross sections of proteins and their complexes: a calibration framework and database for gas-phase structural biology. *Anal. Chem.* **82**, 9557–9565 (2010).
20. Cech, N.B. & Enke, C.G. Practical implications of some recent studies in electrospray ionization fundamentals. *Mass Spec. Rev.* **20**, 362-387 (2001).
